# Chronic Lymphocytic Leukemia Care and Beyond: Navigating the Needs of Long-Term Survivors

**DOI:** 10.3390/cancers17010119

**Published:** 2025-01-02

**Authors:** Stefano Molica, David Allsup

**Affiliations:** 1 Department of Hematology, Hull University Teaching Hospitals NHS Trust, Hull HU3 2JZ, UK; david.allsup@hyms.ac.uk; 2 Centre for Biomedicine, Hull York Medical School, University of Hull, Hull HU6 7RX, UK

**Keywords:** CLL, disease care, beyond treatment, long survivors, long-term monitoring

## Abstract

Chronic lymphocytic leukemia (CLL) treatment has undergone a significant evolution with a shift from historical chemotherapeutic regimens to targeted therapies such as Bruton tyrosine kinase (BTK) and BCL-2 inhibitors. These advancements have been associated with a notable improvement in survival rates with a transformation of CLL into a chronic and manageable condition for most persons with this disease. However, as a consequence of improved outcomes, long-term CLL survivors now face emergent challenges which include a risk of infections, cardiovascular complications, and secondary malignancies. In this changed scenario, holistic models of care are essential to address emergent health risks. Such models of care for CLL patients require a multidisciplinary approach that integrates CLL treatment with the proactive management of frailty, comorbidities, and psychosocial well-being to enhance both survival and quality of life (QoL). CLL predominantly affects older persons, many of whom present with concurrent frailty and comorbidities that may complicate CLL treatment and impact QoL. Comprehensive geriatric assessments (GA) may play a critical role in the identification of persons at a heightened risk of treatment-related toxicity and may help guide rational therapy selection, particularly in very frail persons. In addition to the assessment of hematological responses, the prospective assessment of patient-reported outcomes (PROs) and frailty metrics may offer a more nuanced understanding of the global treatment benefits. A survivorship-focused care model is crucial to address the multifaceted needs of CLL patients with the extension of patient care into the broader domain of long-term health maintenance with associated improvements in QoL.

## Simple Summary

Chronic lymphocytic leukemia (CLL) treatment has evolved from traditional chemotherapy to targeted therapies, such as Bruton tyrosine kinase (BTK) and BCL-2 inhibitors with significant improvements in survival rates and a transformation of CLL into a manageable, chronic condition. However, long-term survivors face an increased risk of infections, cardiovascular complications, and secondary malignancies. This shift in care necessitates the development of holistic survivorship models that incorporate disease treatment with the proactive management of frailty, comorbidities, and psychosocial well-being. Comprehensive geriatric assessments (GAs) are essential in older patients to guide treatment decisions and identify frail multimorbid individuals. The development of CLL survivorship-focused care models, which integrate patient-reported outcomes and frailty metrics, are crucial for the enhancement of both survival and quality of life (QoL).

## 1. Introduction

Chronic lymphocytic leukemia (CLL) affects over 200,000 people in the United States and is a significant public health concern, particularly amongst older persons [1]. Historically, treatment outcomes were constrained by the toxicities and limited efficacy of chemoimmunotherapy with poor prognoses for a proportion of patients [2]. However, over the past decade, the advent of targeted therapies such as inhibitors of Bruton tyrosine kinase (BTK) and BCL-2 has transformed the management of CLL [2]. These innovations have been associated with substantial improvements in survival and shifted the natural history of the disease from an often fatal condition to a chronic, manageable illness [3,4,5].

**Figure 1:**
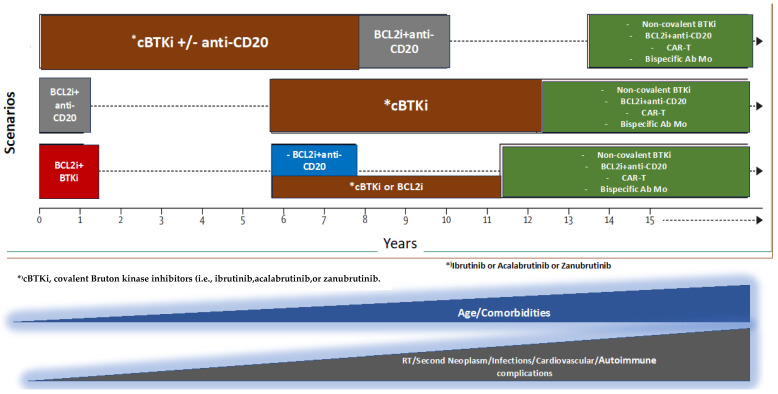
Treatment sequencing in CLL patients. As a consequence of prolonged life-expectancy, patients may experience long-term complications such as the worsening of pre-existing comorbidities, an increased risk of developing second neoplasms, Richter transformation (RT), infections, cardiovascular, and auto-immune complications.

However, despite these therapeutic advancements and improved treatment outcomes, new challenges in patient management have emerged [6,7]. Long-term survivors of CLL may experience a range of secondary health complications such as infections, cardiovascular comorbidities, and psychosocial challenges which are sometimes distinct from predictable treatment-related toxicities [7] (Figure 1). As a result, healthcare providers must now prioritize the development of comprehensive survivorship care plans to address the evolving needs of this patient group.

## 2. The Changing Treatment Landscape of CLL: A Success Story

Before the advent of targeted therapies, patients with CLL had limited treatment options which were primarily based upon chemotherapy regimens [8,9,10]. Agents such as chlorambucil, bendamustine, or fludarabine were commonly used, but these treatments offered only modest improvements in survival and were associated with significant toxicities, including prolonged myelosuppression and increased susceptibility to infection [8,9,10].

The introduction of the anti-CD20 monoclonal antibody rituximab produced a pivotal shift in CLL management [11,12,13,14]. This breakthrough led to the development of chemoimmunotherapeutic approaches, notably the combination of fludarabine, cyclophosphamide, and rituximab (FCR) [11,14]. The FCR regimen produced deep and durable responses in patients who were young and fit enough to tolerate the associated toxicities and this treatment represented a significant advancement in CLL therapy. An updated analysis from the MD Anderson Cancer Center (MDACC) demonstrated that among patients with immunoglobulin heavy-chain variable region gene (*IGHV*) mutated (*IGHV*-M) CLL, those treated with FCR experienced a median progression-free survival (PFS) of 14.6 years. Remarkably, disease progression ten years beyond therapy was rare, which suggests that a subset of patients may achieve what is referred to as a “functional cure” of their CLL [15]. However, despite these promising outcomes, FCR is associated with significant risks, most notably the development of secondary malignancies such as myelodysplastic syndromes (MDS) or acute myeloid leukemia (AML), as well as prolonged immunosuppression with associated increased infection rates [15].

As the CLL treatment landscape has evolved, recent therapeutic developments have prompted a critical reappraisal of the role of FCR, particularly in light of the advent of targeted therapies [16]. A major driver of this reevaluation was the COVID-19 pandemic, which highlighted the vulnerability of immunosuppressed patients [17,18]. FCR-induced myelosuppression is associated with impaired immune responses, reduced vaccination efficacy, and a heightened risk of major infections [18]. These risks underscored the need for alternative treatment strategies that offer efficacy while minimizing immune suppression and the attendant complications.

Recent clinical trials have provided evidence in support of the superiority of targeted therapies over FCR. For example, the US ECOG-ACRIN E1912 trial demonstrated that continuous treatment with the BTK inhibitor (BTKi) ibrutinib, in combination with rituximab, resulted in a progression free survival (PFS) advantage over FCR in young and fit CLL patients, including those with *IGHV*-M disease. The trial also reported a five-year overall survival (OS) benefit for patients treated with ibrutinib-based therapy [19]. Similarly, the GAIA/CLL13 trial highlighted the significantly prolonged PFS with both venetoclax–obinutuzumab (VO) and ibrutinib–venetoclax–obinutuzumab in comparison to chemoimmunotherapy (FCR or BR) and venetoclax–rituximab in previously untreated, fit patients [20]. In clinical practice, FCR remains a reasonable treatment option for a subset of young, fit patients with *IGHV*-M CLL, especially in regions where access to targeted therapies may be limited [21].

Currently, CLL-targeted therapies are the preferred treatment options in the setting of either treatment-naïve or relapsed/refractory CLL [21,22]. The use of BTKi such as ibrutinib, acalabrutinib, and zanubrutinib, or the BCL-2 inhibitor venetoclax, either as a monotherapy or in combination with anti-CD20 monoclonal antibodies, has consistently been associated with superior clinical outcomes when compared to chemoimmunotherapy across various CLL patient populations, including those who are elderly or less fit [19,20,23,24,25,26,27,28,29,30,31,32,33,34,35].

Long-term outcome and safety data for a targeted agent in elderly CLL patients have recently been presented. The final ten year analysis of the landmark RESONATE-2 trial, which compared ibrutinib to chlorambucil in elderly or unfit, previously untreated CLL patients, revealed a median PFS of 8.9 years for ibrutinib-treated patients. In this long-term analysis of the RESONATE-2 trial, the rates of hypertension in the ibrutinib arm during years 8–9 and 9–10 were 28% and 26%, respectively, and 8% and 9% for atrial fibrillation, respectively. During the entire study period, 25% and 33% of patients receiving ibrutinib had any grade adverse events (AEs) leading to dose reduction and discontinuation, respectively. Following dose reduction, 82% of patients had all AEs resolved. Nonetheless, 27% of patients continued ibrutinib treatment for at least ten years, with a median treatment duration of 6.2 years [36].

Such findings underscore the long-term efficacy and tolerability of ibrutinib whilst also highlighting the emergence of a new cohort of CLL patients who survived well beyond their initial diagnosis. Significantly, 70% of patients treated with ibrutinib in the RESONATE-2 trial achieved the 10-year survival milestone. Additionally, combined analyses of relevant clinical trials have demonstrated considerable improvements in OS amongst previously untreated CLL patients, including those aged 65 and older [37]. This progress has substantially narrowed the survival gap between CLL patients and the general population of a similar age, which is a major achievement for modern medicine [38].

## 3. Challenges Faced by Long-Term CLL Survivors

**Figure 2:**
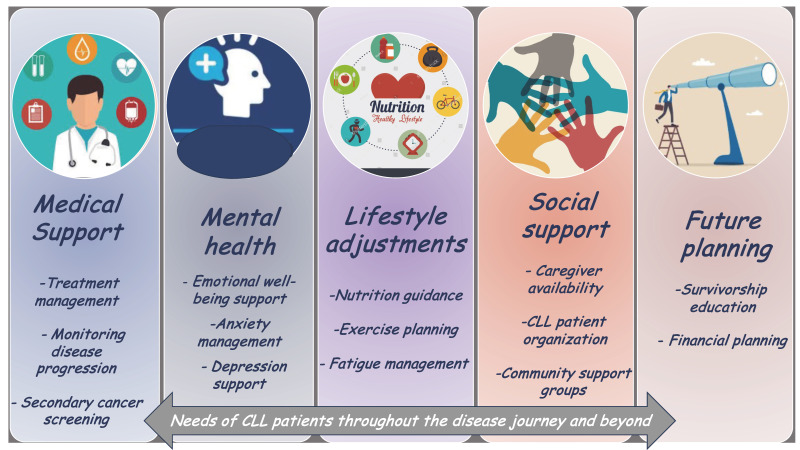
A conceptual graph illustrating the needs of CLL patients throughout their disease journey and beyond, with a particular emphasis on long-term survivors.

As the life expectancy of persons with CLL increases, healthcare providers must address emergent issues in this population such as secondary cancers, cardiovascular complications, and the consequences of ongoing immunosuppression. Additionally, patients may experience treatment-related fatigue, neurocognitive changes, or psychological distress, all of which can significantly impact QoL [7] (Figure 2).

The management of these long-term effects may require a multidisciplinary approach. In addition to the expertise of hematologists and oncologists, the involvement of other healthcare professionals—including infectious disease specialists, cardiologists, and mental health providers—is often necessary. Patient counseling is crucial to help individuals navigate the complexities of living with CLL and its treatments. Support groups and resources for both patients and caregivers can also offer emotional support and practical guidance. In some cases, psychiatric care may be warranted to address anxiety, depression, or other mental health challenges that can arise during the long-term management of CLL.

## 4. The Management of CLL-Related Immune Dysfunction

The heightened risk of infection in CLL is driven by several factors which include disease-induced immune dysregulation, secondary hypogammaglobulinemia, and impaired cell-mediated immunity due to T-cell dysfunction [39]. Serious infections, which may also occur in individuals with monoclonal B-cell lymphocytosis (MBL), are frequently observed in patients with symptomatic CLL. The risk of infection is also present in patients treated with targeted therapies, as these treatments can further compromise immune responses [40,41].

A recent meta-analysis of clinical trials involving CLL patients treated with targeted therapies assessed the risk of infection across various treatment regimens, with infection rates from 13% to 45% of trial participants. The overall incidence of infection was similar between patients treated with BTKi and BCL2 inhibitors (19.8% versus 17.4%). However, amongst those treated with BTKi, the rate of severe infections was increased in those with relapsed or refractory CLL (25.8%) compared to treatment-naïve patients (16.2%) [42]. These findings emphasize the importance of vigilant infection monitoring and the implementation of robust prevention strategies for a subset of CLL patients at a higher risk of developing severe infections while undergoing targeted therapies.

Although vaccine responses are impaired in patients with CLL, immunization remains a critical strategy for the prevention of infections due to the favorable risk–benefit profile of this approach when compared to other infection-preventive measures such as prophylactic antimicrobials and immunoglobulin replacement [43]. Much of the recent understanding in relation to the protective effect of vaccination in persons with CLL is derived from the period during the COVID-19 pandemic [18]. In the large observational European Research Initiative on CLL (ERIC) cohort, CLL patients vaccinated against COVID-19 had lower hospitalization rates and improved OS when compared to unvaccinated individuals. However, vaccination was not identified as an independent factor influencing hospitalization or survival, which indicated that other patient-related factors are likely contributors to CLL patient outcomes. Notably, older age, comorbidities, and CLL-directed treatment were identified as significant risk factors for mortality, whilst vaccination status was not [44].

The European Conference on Infections in Leukaemia (ECIL) group recommends that CLL patients receive an annual single dose of an inactivated influenza vaccine and pneumococcal vaccination, preferably administered before the commencement of CLL-directed therapy. A similar approach is recommended for herpes zoster vaccination [45]. In a recent position paper proposing a model of care for long-term survivors of CLL patients, the strength of the recommendation for influenza, pneumococcal, COVID-19, and varicella herpes zoster vaccines is classified as ‘strong (class 1)’, provided that inactivated vaccines are used [7].

Interestingly, amongst patients treated with ibrutinib, those who had interrupted BTK inhibitor (BTKi) treatment at the time of vaccination exhibited significantly higher antibody titers compared to those who continued their BTKi regimen [46].

Additionally, in CLL long-term survivors who respond to targeted therapies, there is evidence of immunological recovery, which may lead to improved vaccine responses. Data from patients enrolled in the RESONATE and RESONATE2 trials suggest that ibrutinib significantly restored T-cell proliferative capacity, degranulation, and cytokine secretion [47]. Preliminary evidence for the restoration of circulating adaptive and innate immune cells has also been observed in treatment-naïve CLL patients treated with fixed-duration ibrutinib–venetoclax therapy in the CAPTIVATE trial [48]. These findings underscore the need for systematic efforts to improve immune-restorative interventions, especially for long-term survivors of CLL.

An early intervention trial, the PreVent-ACaLL study (NCT03868722), is currently investigating a new scoring system to identify untreated CLL patients who do not meet treatment criteria but are at a high risk of infection. These patients are randomized to either observation or 12 weeks of therapy with acalabrutinib and venetoclax. The primary endpoint is the rate of grade ≥3 infection-free survival at 24 weeks, with a possible extension of the observation period to two years post-enrollment.

Persistent hypogammaglobulinemia, a hallmark of CLL regardless of disease activity, is strongly associated with an increased risk of infection [49]. Immunoglobulin replacement therapy with polyvalent human immunoglobulin G (IgG) is recommended for patients experiencing recurrent severe bacterial infections and those with low IgG levels [50]. A meta-analysis of randomized clinical trials in patients with hematologic malignancies and hypogammaglobulinemia demonstrated that immunoglobulin replacement therapy reduced the incidence of clinically documented infections but did not prevent microbiologically documented infections or infection-related deaths [51].

## 5. The Cardiovascular Complications of BTKis: Risks and Management

CLL primarily affects older adults, with a median age at diagnosis of 72 years [2]. In this demographic group there is a relatively high prevalence of cardiovascular disease (CVD) compared to younger populations. Before the introduction of BTKi therapy, studies indicated that approximately one-third of CLL patients presented with significant CVD at diagnosis and treatment initiation [52,53]. This is concerning, as BTKis may further elevate the risk of cardiovascular events, particularly in those with pre-existing CVD [54]. A recent single-center study suggests that patients with pre-existing CVD have significantly higher odds of experiencing new or worsening atrial fibrillation (AF). This association persisted even after adjusting for comorbidities, the type of BTKi, and baseline medications. These findings underscore the importance of standardized strategies to prevent and promptly detect CV adverse vents (AEs) during BTKi treatment, particularly in patients with pre-existing CVD [55].

Early clinical trials highlighted the association between hypertension and atrial arrhythmias with exposure to the BTKi ibrutinib [56]. In a large cohort of CLL patients (n = 4958), those treated with ibrutinib (6% of the cohort) experienced a 1.91-fold increased risk of stroke and a 3.65-fold increased risk of AF. A pooled analysis of four randomized controlled trials revealed that the incidence of AF was 6.5% in the ibrutinib group compared to 1.6% in the comparator group, with a median time to AF onset of 2.8 months (range: 0.3–26.6 months).

Amongst patients with lymphoid malignancies treated with ibrutinib, the incidence of hypertension was reported to be nearly 72%. Notably, the development of new or worsened hypertension following the initiation of ibrutinib is associated with more than a twofold increase in the risk of other cardiac events [57].

Three phase 3 trials, which compared ibrutinib with the second-generation BTK inhibitors acalabrutinib or zanubrutinib, have been conducted [58,59,60]. Both acalabrutinib and zanubrutinib consistently demonstrated a two- to four-fold reduction in the risk of AF compared to ibrutinib [59,60]. Additionally, acalabrutinib was associated with significantly lower rates of hypertension (9% versus 23% with ibrutinib) [58]. While hypertension rates were similar across patients enrolled in the ALPINE study (24% with zanubrutinib vs. 23% with ibrutinib), they were quantitatively lower with zanubrutinib in the ASPEN trial, which enrolled patients with Waldenström macroglobulinemia (15% vs. 26% with ibrutinib) [59,60]. Interestingly, in the ALPINE trial, mean changes from baseline in the systolic blood pressure over time were generally lower in patients treated with zanubrutinib vs. ibrutinib [61]. In these studies, treatment with antihypertensive medications was linked to a decrease in major adverse cardiovascular events [54].

Ventricular arrhythmias (VAs) and sudden death have emerged as potential class-wide side effects of BTK inhibitors. Ibrutinib is associated with an incidence of VAs of 0.6 to 0.8 per 100 person-years, whereas acalabrutinib and zanubrutinib have reported rates of 0.4 and 0.1 per 100 person-years, respectively. However, the rarity of these events and the limited data on newer agents make these statistics uncertain, emphasizing the need for ongoing monitoring and follow-up [62,63,64].

In the early clinical trials of BTKi therapy, heart failure was not found to be a complication of treatment. However, recent data from pooled long-term follow-ups of several later-phase ibrutinib trials suggest a potential increased risk of heart failure. In these analyses, heart failure was reported in up to 5% of patients, often emerging years after the commencement of treatment [64]. In a large retrospective study involving 860 patients treated with ibrutinib for CLL, a significantly higher risk of developing heart failure was identified compared to chemotherapy treatment (7.7% vs. 3.6%) [65]. Similarly, a pharmacovigilance database study on ibrutinib indicated a more than three-fold increase in the reported odds of heart failure compared to all other drugs in the database [66].

For the second-generation BTKis, limited long-term follow-up precludes an estimation of the risk of heart failure which generally occurs after prolonged drug exposure. In a pooled analysis of 760 patients treated with acalabrutinib, less than 1% experienced any grade of symptomatic congestive heart failure [67]. The evidence concerning heart failure associated with other next-generation agents, including reversible BTK inhibitors, is very limited due to the infrequent reporting of this outcome.

In conclusion, when selecting a BTKi, it is important to take the patient’s cardiac risk profile into account, especially for those with pre-existing cardiovascular risk factors. Nonetheless, the proactive management of modifiable cardiovascular risks and routine monitoring for cardiac toxicity related to the treatment should be implemented for all patients [68]. In this context, transitioning from less selective first-generation BTKis to more selective BTKi options may help refine treatment decisions. Of note, BTKi treatment should be avoided in patients with a history of heart failure, history of ventricular arrhythmias, or uncontrolled hypertension [69].

In animal models, venetoclax has been shown to induce cardiotoxicity through oxidative stress-mediated cardiac inflammation and apoptosis, regulated by the NF-κB and BCL-2 pathways [70]. While cardiac complications are rare in CLL patients treated with venetoclax, a single-center study reported such complications in 20% of patients receiving venetoclax in combination with hypomethylating agents (HMAs) for acute myeloid leukemia (AML) [71]. These findings warrant validation in multicenter, prospective, real-world studies. However, for CLL patients with cardiac comorbidities receiving venetoclax, monitoring for cardiovascular adverse events (CV AEs) is recommended.

## 6. Second Primary Malignancies: Prevalence and Implications for Screening and Long-Term Care

The development of second primary malignancies (SPMs) is associated with increased morbidity amongst CLL patients [72]. A population-based study in the Netherlands demonstrated that CLL patients face a 63% higher risk of developing a SPM compared to an age- and sex-matched population. This increased risk includes both solid tumors and hematological malignancies, with the highest incidence occurring more than five years after CLL diagnosis. The spectrum of SPMs in CLL is broad, and squamous cell carcinoma of the skin, melanoma, lung cancer, colorectal cancer, soft-tissue sarcoma, acute myeloid leukemia (AML), and thyroid cancer are the most commonly observed disorders [73].

In a recent international retrospective study of 19,705 CLL patients, 4134 (21%) developed a second malignancy. Of these, 3088 patients (15.7%) were diagnosed with one or more solid tumors, 834 patients (4.2%) developed a secondary hematological malignancy, and 212 patients (1%) had both types. The median time from CLL diagnosis to the development of a secondary hematological malignancy was 4.7 years. Amongst secondary hematological malignancies, the most prevalent were myelodysplastic syndromes (MDS) (0.4%), AML (0.2%), and myeloma (0.13%). Solid tumors developed at a median of 4.4 years (interquartile range, 2.0–7.6 years) after CLL diagnosis, with non-melanoma skin and prostate cancers being the most common, followed by colorectal (1.9%) and breast cancers (1.7%) [74].

Patients with CLL who developed a SPM had inferior survival when compared to those without a second malignancy. Secondary AML and MDS were associated with the poorest survival outcomes. The FCR regimen was associated with an increased risk of AML and MDS, whereas patients treated exclusively with newer agents such as BTKi or venetoclax were not noted to develop these disorders [72]. These results align with findings from the German CLL Study Group (GCLLSG) registry, which demonstrated a higher-than-expected incidence of hematological SPMs in treated versus untreated CLL patients, likely due to fludarabine-based therapies [75].

Recent studies indicate that in patients treated with BTKis, the occurrence and types of secondary solid tumors are comparable to those observed after chemotherapy or chemoimmunotherapy [76,77]. Nevertheless, prolonged follow-up is required to comprehensively evaluate the effects of targeted treatments for CLL on the risk of developing secondary malignancies [73,74,75,76,77]. Since therapy with targeted agents has improved life expectancy for CLL patients, the long-term risk of developing SPMs will also increase as a function of increased longevity. Therefore, a structured SPM screening program should be offered. These patients should be eligible for annual skin examinations and screening for colon, breast, cervical, and prostate cancers. Smoking cessation counseling and lung cancer screening should also be included in long-term care plans [7].

## 7. Bone Health in CLL: Addressing Fracture Risk and the Potential of BTK Inhibitors

Recent evidence suggests an interaction between bone tissue and CLL cells [78]. Abnormalities of bone metabolism in CLL are associated with elevated serum levels of tumor necrosis factor α, interleukin-6, interleukin-8, and chemokine (C-C motif) ligand 3 (CCL3) [79,80,81,82].

These abnormalities are also associated with the increased expression of the receptor activator of nuclear factor κ-B ligand (RANKL) and vitamin D insufficiency [80,81]. Of note, changes in bone metabolism contribute to a higher risk of axial fragility fractures, even in patients without osteoporosis [78].

A retrospective case-control study using the Surveillance, Epidemiology, and End Results (SEER) registry linked to Medicare, which included 16,344 cases of CLL, reported a 13% incidence of fractures. Notably, the increased fracture risk amongst CLL patients is specific to axial fractures, which supports the concept that cell-cell interactions between leukemic infiltrates and the bone marrow microenvironment may weaken the marrow-rich cancellous bone whilst sparing cortical bone [78].

The increased fracture risk in CLL highlights the need for preventive measures focused on bone health in addition to the monitoring of bone mineral density in both asymptomatic and symptomatic CLL patients. A comprehensive approach could include osteoporosis prevention strategies such as calcium and vitamin D supplementation and regular exercise [7]. Accumulating evidence indicates that vitamin D insufficiency affects approximately 30% of CLL patients. Importantly, vitamin D deficiency has been associated with an increased risk of progression of early stage CLL to a symptomatic phase [83,84]. Finally, for CLL patients with signs of osteoporosis on bone densitometry, the early initiation of antiresorptive therapy is recommended [7].

A retrospective analysis suggests a higher incidence of vertebral fractures in patients treated with ibrutinib [85]. Second-generation BTK inhibitors, such as acalabrutinib and tirabrutinib, inhibit RANKL-induced osteoclast differentiation, thus preventing osteoclastic activity [86,87]. It remains to be demonstrated whether the increasing clinical use of second-generation BTK inhibitors will reduce the risk of bone fractures in CLL patients [88]. Similarly, fixed-duration combinations of venetoclax and ibrutinib, which shorten exposure to ibrutinib, may lower the risk of bone fractures.

## 8. Managing Frailty in CLL

CLL patients are typically above 70 years old and often have comorbidities such as reduced mobility, cognitive impairment, and psychological disorders [89]. These age-related comorbidities are associated with negative health outcomes. In CLL, it has been suggested that prospective evaluation of the markers of frailty should be performed to identify those who are at risk of treatment toxicity [90].

Recent data suggest that targeted therapies for CLL may improve frailty in older adults, which implies that frailty is a dynamic condition with elements of reversibility [91]. HOVON139/GiVe has been the first trial to comprehensively examine geriatric assessments (GA) and frailty in the context of modern targeted CLL therapy. This study, which included frail patients treated with the venetoclax–obinutuzumab combination, demonstrated a significant reduction in the number of geriatric impairments during treatment. Additionally, clinically meaningful improvements were observed in specific subscales of health-related quality of life (HRQoL) [92]. In a retrospective analysis of CLL, for patients older than 80 years old who received ibrutinib, the median PFS and OS were 42.5 and 51.8 months, respectively, and 22.8% experienced a cardiovascular event [93]. The CLL-Frail trial prospectively evaluated the efficacy and safety of acalabrutinib monotherapy in patients ≥80 years of age and/or a FRAIL scale score of ≥3. The first interim analysis of this international phase II study of the evaluation of acalabrutinib in the elderly frail CLL population did not show any unexpected safety signals. Thirty patients were enrolled in the first 12 months of recruitment. At a median observation time of eight months, 21 patients remained on therapy. The reasons for discontinuation were adverse events in five (56%), and death and withdrawn consent in two (22%) patients each, respectively [94].

These findings highlight two key priorities for the clinical trials of elderly frail CLL patients. Firstly, baseline evaluations should incorporate geriatric assessments (GA) to measure frailty and other age-related comorbidities, alongside traditional metrics such as chronological age, Eastern Cooperative Oncology Group Performance Status (ECOG PS), and general illness severity scales. This multi-dimensional approach can provide a clearer picture of an older patient’s health status and suitability for various therapies [95].

Next, tracking outcomes related to frailty and quality of life, in addition to standard hematologic response metrics, is essential. This enables a more holistic understanding of how treatments impact upon patients’ overall well-being and functionality in addition to their cancer status. Alterations in frailty should be regularly assessed and reported, as such changes can be as important to patients’ quality of life as hematologic outcomes [96].

Without these measures, the important benefits of modern CLL therapies could be missed, particularly those that might impact on frailty and sometimes improve overall function in older adults. Furthermore, the incorporation of GA into routine clinical practice could facilitate the identification of patients who may be at a heightened risk of drug-related toxicities [97].

Finally, societal factors such as the absence of a caregiver, distance from the hematology center, and socio-economic factors, may pose barriers to achieving health equity in the management of frail CLL patients. All of these sociodemographic factors should be considered when selecting the most appropriate therapy to ensure improved treatment outcomes [98,99].

## 9. Enhancing the Quality of Life for CLL Patients

Individuals with CLL report lower QoL across most domains when compared to their healthy counterparts [100]. The levels of depression, anxiety, and overall QoL are comparable between patients under watchful waiting and those who are actively treated [101]. However, younger patients managed with a watchful waiting approach often experience depression, diminished emotional and social QoL, and heightened anxiety, whilst older patients tend to report poorer function in physical QoL domains [102].

Routine screening for emotional QoL elements is essential for all patients. Recommended tools to facilitate such assessments include the Geriatric Depression Scale (GDS)-5 and the PROMIS-Anxiety 4-item questionnaire, though alternative screening tools may also be appropriate. Access to psychosocial support from psychologists is critical, irrespective of treatment status [103]. Furthermore, the psychosocial burden on families and caregivers is frequently overlooked and warrants consistent communication and personalized support.

Effective CLL management requires a multidisciplinary, patient- and family-centered approach that addresses both physical and emotional well-being [103]. The shift from chemotherapy to targeted agents has favorably impacted the QoL of persons with CLL [104]. A recent study suggests that ibrutinib-treated patients had better QoL and treatment satisfaction compared to patients receiving chemoimmunotherapy, irrespective of the line of therapy [105]. The improvement in QoL observed with ibrutinib was also noted with second-generation BTKis. Patients with R/R CLL treated with zanubrutinib in the context of the ALPINE trial demonstrated improvements in the global health status compared to ibrutinib-treated patients [106]. Frontline fixed-duration VO improves overall patient reported outcomes (PROs) in older, unfit patients with CLL with and without geriatric impairments. The improvement was based on the patients’ HRQoL, as measured by Patient-Reported Outcomes (PROs). These included assessments of function, depression, cognition, nutrition, physical performance, muscle parameters, and comorbidities, along with evaluations using the European Organization for Research and Treatment of Cancer (EORTC) C30 and CLL17 questionnaires [92].

Finally, anticipated improvements in clinical outcomes rarely fully translate into tangible, patient-centered benefits. Results from an exploratory cross-sectional quantitative survey assessing the quality of relationships between physicians, nurses, and patients, the impact of CLL on daily life, and patient satisfaction with care management suggest significant areas for improvement in these fields [107]. Future studies in CLL should prioritize patient needs, particularly psychosocial and behavioral factors, and focus on improving communication and shared decision-making.

## 10. Conclusions

Advancements in therapies specifically targeting CLL have significantly improved patient outcomes, offering promising prospects for extended survival [37,38,108]. However, it is becoming increasingly clear that improving survival rates for CLL patients will not solely depend on innovation in disease-directed treatments. Instead, the focus must expand to address the competing causes of mortality that affect this patient population [6].

While progress in CLL treatment continues to evolve, there is a growing recognition that CLL patients are at an elevated risk for comorbidities and complications that substantially impact their overall health and survival. These include cardiovascular disease, infections, SPMs and other health conditions not directly related to the leukemia itself [109]. To optimize long-term outcomes, it is essential to shift toward a more comprehensive model of care that goes beyond managing the malignancy alone. This model, grounded in the principles of survivorship, would incorporate proactive monitoring and management of these additional health risks, especially in long-term survivors [7]. Key components of this model would include regular surveillance for comorbidities, preventive healthcare strategies, and individualized interventions aimed at mitigating the risks associated with non-CLL-related factors. This broader focus requires a collaborative, multidisciplinary approach to care, involving oncologists, cardiologists, geriatric specialists, infectious disease experts, and other healthcare providers.

In this model, care would be tailored to each patient’s unique needs, acknowledging the varied challenges they face as CLL survivors. Emphasizing the integration of personalized care plans, which address not only CLL but also the full spectrum of health risks, will be critical in improving both the survival and the quality of life of these patients [7]. By embracing this holistic, patient-centered approach, we can ensure that CLL patients live not only longer, but also healthier lives [110].

Finally, there is a prevalent misconception that CLL has been fully addressed and no longer demands significant research investment. However, none of the currently available targeted therapies provide a definitive cure [111]. As highlighted in this review, the long-term management of aging patients on these treatments is fraught with challenges, including cumulative side effects, the development of drug resistance, and reduced effectiveness over time.

In addition to clinical challenges, targeted therapies impose significant financial burdens. Their prolonged use places immense strain on national healthcare systems, even in wealthy nations capable of sustaining these costs [112]. This dual challenge—balancing clinical efficacy and economic sustainability—reinforces the urgency of research aimed at discovering cost-effective, curative treatments that address both the medical and financial demands of CLL management.

## Data Availability

Data available on request from the authors.
